# Burden of eosinophilic granulomatosis with polyangiitis by disease phase and steroid-sparing effects of biologics: a real-world retrospective study in Europe

**DOI:** 10.1183/23120541.00310-2025

**Published:** 2026-03-02

**Authors:** Jeremiah Hwee, Lynn Huynh, Rafael Alfonso-Cristancho, Wilson da Costa, Mei Sheng Duh, Thanai Pongdee

**Affiliations:** 1 Global Epidemiology, GSK, Mississauga, ON, Canada; 2 Analysis Group, Inc., Boston, MA, USA; 3 Global Real-World Evidence and Health Outcomes Research, GSK, Collegeville, PA, USA; 4 Division of Allergic Diseases, Mayo Clinic, Rochester, MN, USA

## Abstract

**Objectives:**

The aim of the study was to describe insights into real-world characteristics and clinical outcomes of patients with a history of eosinophilic granulomatosis with polyangiitis (EGPA) across Europe, stratified by disease phase and biologics use.

**Methods:**

This was a retrospective, physician-panel chart review study in patients with a history of EGPA from five European countries covering the period January 2015–August 2021 (GSK ID: 214661). Outcomes assessed included: baseline characteristics; treatment patterns; clinical outcomes and healthcare resource utilisation (HCRU), which were stratified *post hoc* by EGPA disease phase and biologics use. Oral corticosteroid (OCS) use was assessed ≤12 months pre- and post-biologics initiation.

**Results:**

Overall, 407 patients were included: disease phase was identified for 381 patients (36 prodromal; 220 eosinophilic; 125 vasculitic); 185 patients received biologics, and 162 had pre-/post-biologics initiation periods identified. Patients in the vasculitic subgroup had the most comorbidities and clinical manifestations, and the greatest proportion of patients with hospitalisation *versus* the eosinophilic or prodromal subgroups. All subgroups had high OCS use (97.2–99.1%). The biologics-exposed subgroup had high comorbidity and HCRU burden. Post- *versus* pre-biologics initiation, OCS use was reduced (0.12 *versus* 0.69 prescriptions per person-year), the proportion of patients experiencing relapses decreased (3.1% (95% confidence interval: 0.4–5.7) *versus* 11.7% (6.8–16.7)) and remissions increased (26.5% (19.7–33.3) *versus* 13.0% (7.8–18.1).

**Conclusion:**

Although the vasculitic subgroup showed the greatest disease severity, all subgroups demonstrated a substantial disease burden. Results also suggest biologics provide OCS-sparing effects and improve disease control in real-world settings.

## Shareable abstract

**All eosinophilic granulomatosis with polyangiitis (EGPA) disease phase subgroups have substantial disease burden; the vasculitic subgroup has more severe disease. Biologics may reduce corticosteroid use and improve EGPA control in a real-world setting.** https://bit.ly/45HyeQQ

## Introduction

Eosinophilic granulomatosis with polyangiitis (EGPA) is a rare and progressive inflammatory disease typically characterised by a combination of blood and tissue eosinophilia, necrotising small-vessel vasculitis, eosinophil-rich granulomatous inflammation and asthma [1, 2]. Patients with EGPA experience multiorgan clinical manifestations that can lead to serious or life-threatening complications [3–6]. In the usual disease course, EGPA develops in three partially overlapping disease phases [1, 5, 7]. The prodromal phase is marked by respiratory tract symptoms such as late onset/worsening asthma and chronic rhinosinusitis; the eosinophilic phase is characterised by blood eosinophilia, eosinophilic tissue infiltration and eosinophil-associated end-organ damage; the vasculitic phase is characterised by the presence of systemic necrotising small-vessel vasculitis and vasculitis-associated manifestations [1, 6–8]. Progression of these phases is not uniform and some patients may not develop overt vasculitic complications.

Treatment for EGPA relies heavily on oral corticosteroids (OCS), often in combination with immunosuppressive/cytotoxic agents [1, 9, 10]. Chronic OCS use and high cumulative exposure are associated with substantial adverse effects, further contributing to disease burden [11–14]. Treatment with approved and emerging biologics, which target type 2 inflammation to induce and then maintain remission, has been associated with OCS-sparing properties [15–19]. Biologics used on or off label to treat EGPA include mepolizumab, an anti-interleukin (IL)-5 monoclonal antibody, benralizumab and reslizumab (which also target the IL-5 pathway), omalizumab (targeting immunoglobulin E), dupilumab (targeting IL-4/IL-13 signalling) and rituximab (targeting CD-20) [15, 17, 19–25]. Of these, mepolizumab and benralizumab are approved for EGPA [26–29], based on data from the pivotal Phase III MIRRA and MANDARA [17] trials [17, 19].

The substantial burden of disease associated with EGPA for both patients and the healthcare system has been demonstrated in prior, mostly database, studies [30–33]. The primary analysis of our longitudinal real-world chart review study supported this burden: in the overall EGPA population almost all patients received OCS, 84.8% experienced clinical manifestations and 36.6% were hospitalised during the follow-up period [34]. Data available on clinical outcomes, characteristics or burden according to EGPA phenotype or disease phase are more limited and have often focused on anti-neutrophil cytoplasmic antibodies (ANCA) status [8, 34–37]. Currently, real-world data on the clinical symptoms, treatment options, burden and outcomes for this rare and heterogeneous disease population remain limited especially for data stratified by prodromal, eosinophilic or vasculitic disease phase or by outcomes according to emerging biologic use. To address these data gaps, we conducted two *post hoc* analyses of the aforementioned real-world European chart review study. The first analysis described demographics, clinical characteristics, treatments and healthcare resource utilisation (HCRU) according to disease phase, while the second analysis described the same outcomes in patients with a history of EGPA treated with biologics and also examined OCS use and clinical outcomes pre- and post-biologic initiation.

## Methods

### Study design

The parent study was a retrospective, non-interventional, longitudinal, real-world, physician panel-based chart review study (GSK ID: 214661) of patients diagnosed with EGPA across five European countries (Germany, Italy, France, Spain, UK) [34]. Approximately 40 physicians per country with a specialty in rheumatology, pulmonology, allergy or immunology were recruited. Eligible physicians were required to have access to the complete medical records of ≥1 eligible patient with EGPA, and to be their primary healthcare provider. Physicians abstracted anonymised data using standardised and electronic case report forms (eCRFs). For physicians with multiple eligible patients, mechanisms were put in place to prevent bias in patient selection [34]. Physicians were blinded to the study sponsor's identity and vice versa.

### Study population

Participating physicians identified eligible patients from among those who had a first clinical visit for any reason with the physician between January 2015 and December 2019 (patient identification window). The index date was the date of the first clinical visit within the patient identification window. Eligible patients had a physician-confirmed diagnosis of EGPA, were aged ≥12 years at EGPA diagnosis and had ≥1 year of data from the index date to the end of the follow-up (EOF) period that was accessible to the physician (unless follow-up ended earlier due to death). Patients may have already been diagnosed with EGPA or were newly diagnosed at the index date visit. The diagnosis date was the date of first EGPA diagnosis.

### Outcomes and assessments: post hoc disease phase and biologics-exposed subgroups

Demographic and baseline disease characteristics were obtained from patient charts from the pre-index period (between EGPA diagnosis and the index date for patients diagnosed before index; these characteristics were also collected for patients diagnosed at index). Disease phase (prodromal, eosinophilic or vasculitic) at the index date was recorded by physicians based on assessments made before or on the index date. EGPA clinical manifestations, including the presence of asthma airway reversibility (*i.e.* improvement in response to a short-acting bronchodilator such as albuterol), clinical outcomes (remission and relapse) and HCRU were assessed from the index date until EOF. Comorbid conditions were assessed from EGPA diagnosis until EOF. Treatment use was assessed from EGPA diagnosis until EOF (unless otherwise stated) for OCS (including dosage), immunosuppressant/cytotoxic agents, biologics and other therapies. Ongoing treatments at EOF were also documented.

Remission was defined by physician assessment. The most common definitions reported were a Birmingham Vasculitis Activity Score of 0 or an OCS dose of ≤4 mg·day^−1^. Relapse was defined as a recurrence or worsening of EGPA symptoms requiring an increase in OCS dose, an increase/change in immunosuppressive therapy dose or hospitalisation; other physician-definitions were also included. The above outcomes, relapse-free survival time and overall survival time were summarised for prodromal, eosinophilic and vasculitic subgroups.

For patients diagnosed with EGPA before the index date, a pre-index period was utilised from which patient demographics and baseline clinical characteristics were identified. Follow-up included the period from the index date to the earliest occurrence of death, loss to follow-up or end date of chart abstraction. The last date of follow-up was August 2021 (supplementary figure S1A). This manuscript describes *post hoc* subgroup analyses by EGPA disease phase and biologic treatment exposure, with disease phase subgroups based on the assignment (prodromal, eosinophilic, vasculitic or unknown) recorded in the eCRF. The biologic use subgroup was defined *post hoc* as patients with a record of receiving biologic treatment between diagnosis and EOF. For patients who had ≥1 non-missing dates for ≥1 biologics administration recorded, a date of biologics initiation, and pre- and post-biologics periods were defined (supplementary figure S1B).

For the biologic-exposed subgroup, patient demographics, disease characteristics, comorbidities and HCRU were summarised. Additionally, where a date of biologics administration was available, details of OCS use, clinical manifestations and clinical outcomes for patients treated with biologics were summarised for the periods ≤12 months pre- and post-biologic initiation. The pre-biologics period was defined as 12 months before and included the initiation of biologics. Only events and person-years after EGPA diagnosis were included*.* The post-biologics period was defined as 12 months after the initiation of biologics or until death or EOF (*i.e.* patients were censored if they died or had <12 months of follow-up period after biologics initiation).

### Sample size and statistical analyses

No formal sample size calculation was performed. All outcomes were summarised using descriptive statistics as n (%), mean±sd or median (interquartile range (IQR)), and no comparative testing was conducted. The number of relapses was annualised and reported per-patient-per-year (PPPY). As the incidences of relapse and death were observed to be <50%, the median survival times could not be estimated so restricted mean survival times (RMSTs) were reported. Real-world relapse-free survival and overall survival were assessed over a 6-year period following the diagnosis date *via* Kaplan–Meier analysis. Data were analysed using SAS Enterprise Guide Version 7.15 (SAS Institute Inc., Cary, NC, USA).

### Ethics

This study complied with all applicable laws regarding subject privacy. Results were obtained from aggregate analyses that omitted patient identification, with no direct patient contact or primary collection of individual patient data so ethics approval and informed consent were not required.

## Results

A total of 204 physicians were recruited from targeted specialties, including rheumatology (n=89, 43.6%), pulmonology (n=76, 37.3%), allergy (n=26, 12.7%) and immunology (n=13, 6.4%). Physicians were evenly distributed between the five study countries. Overall, 407 patients were eligible for inclusion; of these, disease phase information was available for 381 patients: prodromal phase (n=36), eosinophilic phase (n=220) and vasculitic phase (n=125). The remaining 26 patients had unknown disease phase. Overall, 185 patients received biologic therapy between diagnosis and EOF (supplementary figure S1B).

### EGPA disease phase subgroup analysis

#### Patient demographics and clinical characteristics

Patient demographics across disease phases were broadly similar. In all countries, at any timepoint from diagnosis, most patients were in the eosinophilic phase and the fewest patients were in the prodromal phase. Patients in the prodromal phase were more common in the UK, France and Spain, whereas the vasculitic phase was more common in Germany and Italy. Patients in the prodromal phase were slightly younger (median age) than patients in the eosinophilic or vasculitic phase, while patients in the vasculitic phase exhibited more comorbidities and a higher median blood eosinophil count *versus* other disease phases (table 1; supplementary table S1). Compared with the other disease phases, a larger proportion of patients with prodromal EGPA were former smokers, and asthma airway reversibility was less common in patients with vasculitic EGPA. Diagnostic tests and assessments varied across the disease phases. Tests for ANCA, imaging scans of affected organs, biopsy to detect extravascular eosinophils and tests for neuropathy were all most common in the vasculitic phase. For all disease phases, most patients were under the care of a pulmonologist or rheumatologist, while 20.5% of patients with eosinophilic phase EPGA were under the care of an allergist (table 1). Patient demographics and disease characteristic stratified by physician speciality are presented in supplementary table S2.

**TABLE 1:** Baseline patient demographics and disease characteristics across EGPA disease phase

Patient demographics and disease characteristics	Overall	Prodromal	Eosinophilic	Vasculitic
**Patients, n**	407	36	220	125
**Patient country, n (%)**				
France	81 (19.9)	9 (25.0)	47 (21.4)	21 (16.8)
Germany	80 (19.7)	4 (11.1)	40 (18.2)	33 (26.4)
Italy	80 (19.7)	4 (11.1)	44 (20.0)	28 (22.4)
Spain	85 (20.9)	9 (25.0)	48 (21.8)	20 (16.0)
UK	81 (19.9)	10 (27.8)	41 (18.6)	23 (18.4)
**Age at EGPA diagnosis years**				
Median (IQR)	44.5 (32.8–53.7)	41.2 (34.1–54.2)	45.1 (33.3–52.9)	44.1 (32.2–54.1)
≥18 years of age, n (%)	383 (94.1)	33 (91.7)	206 (93.6)	118 (94.4)
**Male, n (%)**	231 (56.8)	19 (52.8)	127 (57.7)	73 (58.4)
**EGPA diagnosis, n (%)**				
Before study (2014 or before)	48 (11.8)	4 (11.1)	25 (11.4)	13 (10.4)
During study (2015–2019)	359 (88.2)	32 (88.9)	195 (88.6)	112 (89.6)
**Disease duration (between EGPA diagnosis and EOF), median (IQR**)	2.5 (1.8–4.1)	2.4 (1.8–3.8)	2.4 (1.8–3.9)	2.8 (1.9–4.4)
**Physician speciality, n (%)**				
Allergy	60 (14.7)	5 (13.9)	45 (20.5)	6 (4.8)
Immunology	28 (6.9)	5 (13.9)	13 (5.9)	10 (8.0)
Rheumatology	165 (40.5)	12 (33.3)	73 (33.2)	70 (56.0)
Pulmonary	154 (37.8)	14 (38.9)	89 (40.5)	39 (31.2)
**Comorbidities, n (%)**				
Vasculitis	197 (48.4)	8 (22.2)	87 (39.5)	92 (73.6)
Hypertension^#^	163 (40.0)	10 (27.8)	81 (36.8)	61 (48.8)
Anxiety or depression	140 (34.4)	12 (33.3)	70 (31.8)	54 (43.2)
Lower respiratory disease(s)^¶^	77 (18.9)	6 (16.7)	42 (19.1)	29 (23.2)
Osteoporosis^#^	72 (17.7)	6 (16.7)	34 (15.5)	27 (21.6)
Glomerulonephritis	69 (17.0)	1 (2.8)	26 (11.8)	39 (31.2)
Obesity	68 (16.7)	6 (16.7)	34 (15.5)	23 (18.4)
Diabetes^#^	35 (8.6)	5 (13.9)	15 (6.8)	13 (10.4)
Rheumatoid arthritis	20 (4.9)	0 (0.0)	9 (4.1)	10 (8.0)
Liver disease	11 (2.7)	1 (2.8)	6 (2.7)	4 (3.2)
Other	22 (5.4)	2 (5.6)	11 (5.0)	8 (6.4)
Cancer (any)^+^	8 (2.0)	0 (0.0)	4 (1.8)	4 (3.2)
**Blood eosinophils**				
Patients with data available, n (%)	364 (89.4)	33 (91.7)	196 (89.1)	115 (92.0)
Count cells·µL^−1^, median (IQR)	1500 (600–3300)	1200 (120–2000)	1500 (560–3300)	1800 (1000–5000)
**Proportion of patients with asthma, n (%)**	299 (73.5)	21 (58.3)	164 (74.5)	95 (76.0)
**Time from asthma diagnosis to EGPA diagnosis, years, median (IQR)^§^**	1.8 (0.2–5.6)	3.0 (0.3–8.9)	1.5 (0.1–4.3)	2.9 (0.1–6.1)
**Length of follow-up years, mean±sd**	2.7±1.4	2.6±1.4	2.7±1.5	2.8±1.4

COPD: chronic obstructive pulmonary disease; EGPA: eosinophilic granulomatosis with polyangiitis; EOF: end of follow-up; IQR: interquartile range. ^#^: potentially steroid-related; ^¶^: other than asthma and COPD; ^+^: reported cancer types included: lung (3), breast (1), colon (1), leukaemia (1), rectum (1), skin (1); ^§^: among 154 patients who had asthma diagnosed before EGPA.

#### Treatment use

Patients in the vasculitic phase received the highest mean±sd number of distinct therapies (4.4±1.7) *versus* prodromal (3.8±2.1) and eosinophilic phases (3.7±1.8). Notably, 86.4% of patients with vasculitic EGPA received ≥3 distinct EGPA therapies *versus* 63.9% and 71.4% of those in the prodromal and eosinophilic phases, respectively (supplementary table S3).

**FIGURE 1:**
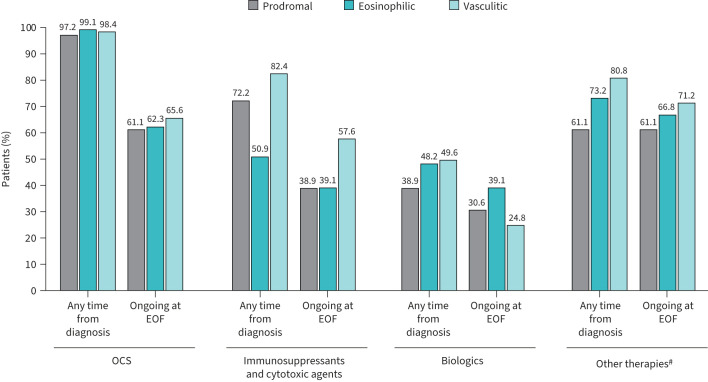
Treatment use at any time since diagnosis and at end of follow-up (EOF) by eosinophilic granulomatosis with polyangiitis (EGPA) disease phase. OCS: oral corticosteroids. ^#^: other treatments for the control of EGPA-related clinical manifestations.

OCS were the most common treatment across all disease phases and were used by almost all patients (n=35, 97.2%; n=218, 99.1%; and n=123, 98.4% in prodromal, eosinophilic and vasculitic phases, respectively; figure 1). The mean±sd maximum daily dose for maintenance OCS was highest in patients in the prodromal subgroup (37.8±19.5 mg) *versus* the eosinophilic (29.1±19.6 mg) or vasculitic (29.8±19.7 mg) subgroups (supplementary table S3). Over half of patients in each subgroup were receiving ongoing OCS treatment at EOF (figure 1).

A greater proportion of patients in the eosinophilic and vasculitic phases compared with the prodromal phase received biologic therapy (n=106, 48.2%; n=62, 49.6% and n=14, 38.9%, respectively). None of the biologics used had been approved in Europe for the treatment of EGPA at the time of the follow-up period. Mepolizumab was the most commonly used biologic in the eosinophilic phase (n=46, 20.9%) and rituximab was the most commonly used biologic in the prodromal and vasculitic phases (n=6, 16.7% and n=33, 26.4%, respectively; supplementary table S3). At EOF, ongoing biologics use was lower in the vasculitic subgroup (n=31, 24.8%) compared with prodromal (n=11, 30.6%) and eosinophilic phases (n=86, 39.1%; figure 1). Mepolizumab remained the most common ongoing biologic therapy at EOF among the eosinophilic subgroup (n=41, 18.6%), while rituximab was most common in prodromal and vasculitic subgroups (n=6, 16.7% and n=14, 11.2%, respectively). Irrespective of the disease phase, there was typically a >1 year gap between EGPA diagnosis and initiation of biologic treatments (supplementary table S3).

#### Clinical manifestations

**FIGURE 2:**
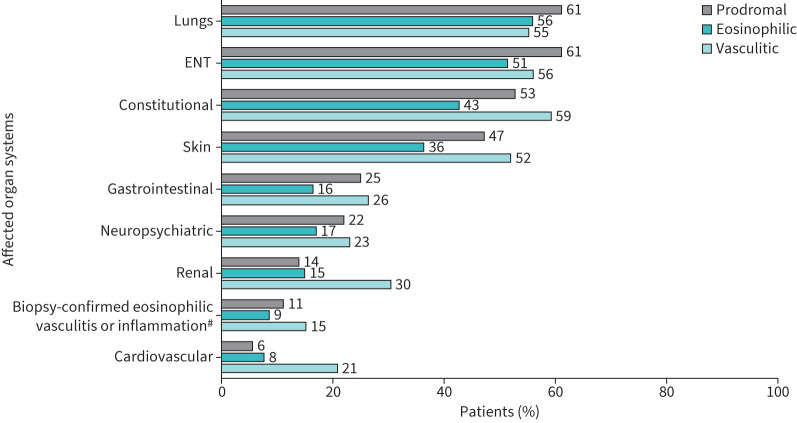
Clinical manifestations by organ involvement across eosinophilic granulomatosis with polyangiitis (EGPA) disease phases. ENT: ear, nose and throat. ^#^: biopsy sites were skin (20), lung (9), kidney (3), muscle (2), alveoli (1), lower extremity (1), intestine (1), peripheral nerve (1) and unknown (4).

Patients in the vasculitic phase had a higher median (IQR) number of distinct clinical manifestations (4.0 (2.0–7.0)) compared with those in the prodromal (3.0 (1.5–6.0)) and eosinophilic phases (3.0 (1.0–5.0)). Similarly, the proportion of patients with ≥6 distinct clinical manifestations was greatest in the vasculitic phase (36.8%) *versus* the prodromal (27.9%) and eosinophilic phases (21.8%). The lungs, ear, nose and throat (ENT) and skin were identified as the most commonly affected organ systems across all disease phases (supplementary table S4). Additionally, constitutional manifestations or symptoms were frequently observed in all phases but were highest in the vasculitic subgroup (n=74, 59.2%; figure 2). Renal and cardiovascular system manifestations were more frequent among the vasculitic subgroup than among prodromal and eosinophilic subgroups.

#### Clinical outcomes

The proportion of patients achieving remission was greatest among patients in the vasculitic phase (n=79, 63.2%), followed by the eosinophilic (n=133, 60.5%), then the prodromal phase (n=17, 47.2%). However, among the patients achieving remission, the median (IQR) duration of time in remission was longer in the prodromal subgroup (16.0 (7.5–28.5) months) than in the eosinophilic or vasculitic subgroups (10.6 (4.6–20.5); 13.4 (7.0–24.7) months, respectively; table 2). Patients in the vasculitic phase were more likely to experience a relapse (n=36, 28.8%) than patients in the prodromal (n=8, 22.2%) or eosinophilic phase (n=32, 14.5%). Among patients who experienced a relapse, the annualised mean number of relapses PPPY was similar between patients in the vasculitic and eosinophilic phases, and lower in the prodromal phase. For relapse-free survival, patients in the vasculitic phase had a lower 6-year RMST (4.61 years) *versus* patients in the prodromal (4.84 years) and eosinophilic phases (5.26 years; supplementary figure S2). 11 deaths were reported during the study; overall survival rates were similar across different disease phases (supplementary figure S3).

**TABLE 2:** Clinical outcomes across EGPA disease phases

Clinical outcomes	Prodromal	Eosinophilic	Vasculitic
**Patients, n**	36	220	125
**Patients who experienced remission^#^, n (%)**	17 (47.2)	133 (60.5)	79 (63.2)
Cumulative duration of remission(s) months, median (IQR)^¶^	16.0 (7.5–28.5)	10.6 (4.6–20.5)	13.4 (7.0–24.7)
Time from diagnosis to first remission months, median (IQR)	14.3 (7.9–31.6)	15.9 (8.1–27.2)	12.5 (6.9–24.2)
**Patients who experienced a relapse^+^, n (%)**	8 (22.2)	32 (14.5)	36 (28.8)
Annualised number of relapses (among patients with a relapse), PPPY, median (IQR)	0.4 (0.3–0.6)	0.6 (0.3–1.0)	0.6 (0.4–1.0)

EGPA: eosinophilic granulomatosis with polyangiitis; IQR: interquartile range; PPPY: per-patient-per-year; BVAS: Birmingham Vasculitis Activity Score; OCS: oral corticosteroids. ^#^: remission was defined by the physician; definitions used included BVAS=0, OCS dose ≤4 mg·day^−1^ and other. ^¶^: for patients who had >1 remission, the duration of remission was calculated as the sum of durations of remission for all reported remissions. ^+^: relapse was defined as an increase in OCS dose, an increase/change in dose of immunosuppressive therapy, or hospitalisation; indication of relapse based on other definitions was also included.

#### Healthcare resource utilisation

Compared with the prodromal and eosinophilic phases, patients in the vasculitic phase had the highest rate of hospitalisations (57.6% *versus* 33.3% and 27.3%) and outpatient visits (87.2% *versus* 75.0% and 84.1%, respectively). In contrast, patients in the prodromal phase had the highest proportion of emergency room (ER) visits *versus* the eosinophilic and vasculitic phases (44.4% *versus* 19.1% and 32.8%, respectively; supplementary table S5).

### Biologics use subgroup analysis

#### Patient demographics, clinical characteristics and comorbidities

Overall, 45.5% of patients received biologics. Mepolizumab and rituximab were the most commonly prescribed biologics (n=74, 40.0% for each treatment). Others were benralizumab (n=26, 14.1%), omalizumab (n=18, 9.7%), reslizumab (n=16, 8.6%) and dupilumab (n=1, 0.5%) (table 3 and supplementary table S6). Some patients received multiple biologics over the period from EGPA diagnosis to EOF. There was a high prevalence of comorbidities among patients who received biologics, including anxiety or depression (n=87, 47.0%), hypertension (n=81, 43.8%) and osteoporosis (n=39, 21.1%).

**TABLE 3:** Demographics, clinical characteristics and comorbidities of patients who received biologics (n=185)

**Biologics used^#^, n (%)**	
Mepolizumab	74 (40.0)
Rituximab	74 (40.0)
Benralizumab	26 (14.1)
Omalizumab	18 (9.7)
Reslizumab	16 (8.6)
Dupilumab	1 (0.5)
**Age at EGPA diagnosis years**	
Median (IQR)	44.2 (33.6–52.8)
**Male, n (%)**	98 (53.0)
**Patient country, n (%)**	
France	43 (23.2)
Germany	17 (9.2)
Italy	55 (29.7)
Spain	43 (23.2)
UK	27 (14.6)
**Comorbidities, n (%)**	
Asthma	147 (79.5)
Anxiety or depression	87 (47.0)
Hypertension^¶^	81 (43.8)
Osteoporosis^¶^	39 (21.1)
Lower respiratory disease^+^	36 (19.5)
Glomerulonephritis	36 (19.5)
Obesity	36 (19.5)
Diabetes^¶^	21 (11.4)
Rheumatoid arthritis	13 (7.0)
Cancer	8 (4.3)
Liver disease	5 (2.7)
Other	12 (6.5)
**Disease duration (between EGPA diagnosis and EOF), median (IQR)**	2.6 (1.9–4.4)
**Year of diagnosis, n (%)**	
2014 or before	23 (12.4)
2015–2019	162 (87.6)
**Disease phase, n (%)^§^**	
Prodromal	14 (7.6)
Eosinophilic	106 (57.3)
Vasculitic	62 (33.5)
Unknown	3 (1.6)
**Patients with confirmed asthma diagnosis date before EGPA diagnosis, n (%)**	76 (41.1)
Time from asthma diagnosis to EGPA diagnosis, years, mean±sd^ƒ^	3.4±5.7
**Length of follow-up years, mean±sd**	2.8±1.5

EGPA: eosinophilic granulomatosis with polyangiitis; EOF: end of follow-up. ^#^: biologics were not mutually exclusive; some patients received multiple different biologics between diagnosis and EOF. Mepolizumab is currently the only approved biologic for EGPA in the EU. ^¶^: potentially steroid-related. ^+^: other than asthma or COPD. ^§^: disease phase was assessed on or before index date. ^ƒ^: among the 76 patients in the biologics-exposed group who had a reported asthma diagnosis date before EGPA diagnosis.

#### Healthcare resource utilisation

Among patients who received biologics, 91 (49.2%) required EGPA-related hospitalisation, with a mean±sd length of stay of 8.6±7.4 days. The proportion of patients who made ER visits and outpatient visits was 34.1% and 88.1%, respectively (supplementary table S7).

#### Post- versus pre-biologics initiation outcomes

OCS use was reduced in the ≤12 months post-biologics initiation period *versus* the ≤12 months pre-biologics period, assessed by reductions in both the number of patients with ≥1 OCS prescription (n=14, 8.6% (95% confidence interval (CI) 4.3–13.0) *versus* n=70, 43.2% (95% CI 35.6–50.8)) and the annualised rate of OCS prescriptions (0.12 *versus* 0.69 PPPY; table 4).

**TABLE 4:** Clinical manifestations, clinical outcomes and OCS use in patients with a history of EGPA receiving biologics

	Patients with non-missing date for ≥1 biologics administration^#,¶^	
	≤12 months pre-biologic initiation	≤12 months post-biologic initiation
**OCS use**		
≥1 OCS prescription, n (%), 95% CI	70 (43.2), 35.6–50.8	14 (8.6), 4.3–13.0
Annualised rate of OCS prescription PPPY	0.69	0.12
**Clinical manifestations, n (%), 95% CI**		
Severe asthma	28 (17.3), 11.5–23.1	11 (6.8), 2.9–10.7
Shortness of breath	22 (13.6), 8.3–18.9	13 (8.0), 3.8–12.2
Fatigue	18 (11.1), 6.3–16.0	16 (9.9), 5.3–14.5
Myalgia/arthralgia	15 (9.3), 4.8–13.7	6 (3.7), 0.8–6.6
Nasal polyposis	14 (8.6), 4.3–13.0	5 (3.1), 0.4–5.7
Allergic rhinitis	13 (8.0), 3.8–12.2	8 (4.9), 1.6–8.3
Lung infiltrates	12 (7.4), 3.4–11.4	9 (5.6) (2.0–9.1
Paranasal sinusitis	9 (5.6), 2.0–9.1	8 (4.9), 1.6–8.3
Peripheral neuropathy	7 (4.3), 1.2–7.5	5 (3.1), 0.4–5.7
Cardiovascular	5 (3.1), 0.4–5.7	7 (4.3), 1.2–7.5
Glomerulonephritis	4 (2.5), 0.1–4.9	4 (2.5), 0.1–4.9
Mononeuritis	4 (2.5), 0.1–4.9	2 (1.2), 0–2.9
**Clinical outcomes, n (%)**, **95% CI**		
Relapse	19 (11.7), 6.8–16.7	5 (3.1), 0.4–5.7
Remission	21 (13.0), 7.8–18.1	43 (26.5), 19.7–33.3

OCS: oral corticosteroids; EGPA: eosinophilic granulomatosis with polyangiitis; PPPY: per-patient-per-year; EOF: end of follow-up. ^#^: n=162. ^¶^: in pre-biologics period, only OCS prescriptions and person-years after EGPA diagnosis were included. In post-biologics period, patients were censored if they died or had <12 months of follow-up period after biologics initiation. OCS prescriptions between EGPA diagnosis and EOF were assessed.

Clinical manifestations were reduced or unchanged following biologic initiation, with notable reductions in respiratory and ENT manifestations and myalgia/arthralgia (table 4). There was a decrease in the number of patients experiencing a relapse in the ≤12 months post-biologics initiation period compared with the ≤12 months pre-biologics period (n=5, 3.1% (95% CI 0.4–5.7) *versus* n=19, 11.7% (95% CI 6.8–16.7)), and a greater proportion of patients achieving remission (n=43, 26.5% (95% CI 19.7–33.3) *versus* n=21, 13.0% (95% CI 7.8–18.1)).

## Discussion

These *post hoc* subgroup analyses of real-world European data offer novel insights into the distinct characteristics, treatment needs and outcomes for patients with a history of EGPA according to disease phase and use of biologics. Notable differences between disease phases were observed, with the vasculitic phase presenting more severe symptoms than the eosinophilic or prodromal phases. A varying prevalence of disease phases across countries was observed, which may be attributable to differences in patient populations. There was a high OCS requirement across all disease phases. Treatment with immunosuppressants and biologics at any time during the study period was more common among patients in the vasculitic phase compared to the prodromal and eosinophilic phases. However, relative to the prodromal and eosinophilic phases, fewer patients in the vasculitic phase were indicated to have remained on biologics at the EOF.

In the subgroup analysis of patients who received biologics, the time from asthma diagnosis to EGPA diagnosis was shorter than in patients in the overall group. Patients who received biologics also experienced a substantial burden from comorbidities, including conditions commonly associated with OCS use, such as anxiety or depression, diabetes and osteoporosis [11–13]. Many patients who received biologics also required tests for complications or monitoring of adverse effects from using immunosuppressive medications, highlighting the substantial OCS-related burden. Additionally, high HCRU was observed in this subgroup, with almost half of patients requiring hospitalisation, indicating they represent patients with severe EGPA. Nonetheless, OCS-sparing effects including a 5-fold reduction in annualised OCS prescription rate per patient and improved disease control were observed post-initiation of biologics, providing real-world evidence of their benefit.

Initiation of biologics typically occurred >1 year after diagnosis; mepolizumab and rituximab were the most commonly used biologics. Not all patients who started biologic therapy in our study were receiving OCS at the time, suggesting that many biologics (other than rituximab) may have been initiated to manage asthma or rhinosinusitis rather than systemic manifestations of EGPA. However, we did not collect data on the reason for biologic initiation so we cannot confirm this speculation. While no biologics were approved for EGPA in Europe at the time of this study, mepolizumab was approved for patients with severe eosinophilic asthma during the patient identification period [27], and real-world studies have demonstrated that mepolizumab improves both EGPA and asthma outcomes [38, 39]. Since then, mepolizumab and benralizumab have both been approved specifically for the treatment of EGPA in Europe [27, 28]. Other biologics (omalizumab, reslizumab, dupilumab) taken by patients in this study are not approved for EGPA, but are approved for severe asthma associated with type 2 inflammation [40–42].

Data for mepolizumab use in EGPA are well established (having been approved in EGPA in Europe since 2021 and in the USA since 2017) [26, 27]. The phase III MIRRA trial demonstrated that patients with relapsing/refractory EGPA receiving mepolizumab had lower relapse rates, spent more time in remission and were able to reduce OCS use compared with placebo [19]. In addition, *post hoc* analyses of the MIRRA trial showed clinical and OCS-sparing benefits with mepolizumab treatment compared with placebo, regardless of baseline characteristics or vasculitic phenotype [16, 18, 37, 43]. More recently, in the head-to-head MANDARA trial benralizumab showed statistically non-inferior effects compared to mepolizumab across outcomes including remission achievement, relapse and OCS reduction [17]*.* However, a gap still exists in understanding the real-world use and effectiveness of biologics, particularly across different patient subgroups. The MARS observational study of patients with EGPA in Japan indicated that mepolizumab was well tolerated, facilitated OCS dose reduction and improved disease control, with lower rates of EGPA-related relapse/hospitalisations and asthma exacerbations *versus* pre-treatment [38]. A retrospective European study on the efficacy and safety of rituximab, mepolizumab and omalizumab in refractory/relapsing EGPA found rituximab may be effective for treating vasculitic relapses; mepolizumab was reported as effective and safe for patients who also had glucocorticoid-dependent asthma, whereas omalizumab had limited efficacy [44]. In observational studies, rituximab has been shown to induce remission in 36–100% of patients, with relapse rates of 10–54% (median 33%) [23]. However, in the Phase III randomised controlled REOVAS trial rituximab failed to demonstrate a difference in remission induction *versus* conventional therapy in EGPA [45]. Both observational studies and long-term follow-up of the Phase III trial suggest long-term outcomes with rituximab may be better for ANCA-positive patients than ANCA-negative patients [23, 46].

Historically, the 2009 European Alliance of Associations for Rheumatology (EULAR) guidelines recommended only OCS and immunosuppressants for primary small and medium vessel vasculitis, potentially explaining the widespread use of OCS across all disease phases in this study. The 2016 amendment to the guidelines introduced biologic treatment, such as rituximab (approved for use in patients with granulomatosis with polyangiitis and microscopic polyangiitis) for certain ANCA-associated vasculitis subsets, including EGPA, which occurred within this study's timeframe (2015–2019) [47]. Updates to the EULAR guidelines (2022) included more specific recommendations for biologics like mepolizumab and rituximab in EGPA [10]. Mepolizumab is recommended for induction/maintenance of remission in patients with relapsing/refractory disease (without active organ-threatening or life-threatening disease) and can be used for maintenance of remission (in organ-threatening or life-threatening disease). Rituximab can also be used in the latter case and in new-onset or relapsing EGPA with organ-threatening/life-threatening manifestations to induce remission [1, 10]. The relatively recent nature of these updates may explain the observed underutilisation of biologics in this study. The high OCS usage observed in this study for all patients conforms with the current EULAR guidelines [10]. For more severe new-onset or relapsing EGPA, adding immunosuppressants to OCS treatment is advised. This aligns with the higher frequency of immunosuppressant prescriptions in vasculitic patients observed in this study, likely due to this disease stage being associated with more severe sequelae and relapses [7, 8].

These *post hoc* subgroup analyses, derived from a large cohort of patients with a history of EGPA, address the notable gap in real-world data on EPGA disease burden, treatment patterns, clinical outcomes and HCRU, stratified by disease phase or biologics use. The inclusion of multiple European populations is a strength of the study and enhances the generalisability of these findings, offering valuable insights into EGPA management across various settings.

Limitations of this study include the potential for bias inherent in observational research, as well as the absence of predefined criteria for EGPA diagnosis and for different disease stages provided to physicians. The categorisation into prodromal, eosinophilic or vasculitic phases was based on the clinical judgement of each physician, which may have varied owing to a lack of consensus diagnostic criteria for EGPA and the difficulties in distinguishing between prodromal and eosinophilic EGPA. The timepoint of disease classification was not specified and may have been at any time from diagnosis and the classification. The classification into severe (life- or organ-threatening) and non-severe EGPA, as suggested by current guidelines, was not assessed in this study. The design of our eCRF may have led to an underestimation of the proportion of patients with pulmonary manifestations of EGPA. First, we considered asthma to be a substantial component in the diagnosis of EGPA and, for that reason, physicians were not asked to record asthma as a lung-specific clinical manifestation. Second, physicians were asked to record comorbid conditions diagnosed between EGPA diagnosis and EOF, so patients with an asthma diagnosis prior to EGPA diagnosis may not have had asthma recorded as a comorbid condition. Similarly, since physicians relied on patient's medical records to complete the eCRF, some of the data may have been incomplete, particularly if the physician who participated in the study was not the one who made the EGPA diagnosis, potentially leading to underestimation of some baseline characteristics (*e.g.* number of patients who had biopsies). The primary study was not originally designed to assess the impact of biologics, and some patients received multiple different biologics over the course of the study; therefore, physicians should use caution when applying these findings to clinical practice. These aspects highlight the need for more standardised diagnostic criteria in EGPA research and careful interpretation of the implications of biologic therapies. Further research is needed to fully understand the real-world impact of specific biologic therapies used to treat EGPA.

These *post hoc* subgroup analyses of a large, retrospective, physician-panel chart review of patients with a history of EGPA across five European countries examine how disease phase may influence presentation and how biologics are used in this rare and varied disease area. These analyses highlight the long wait patients may experience to receive diagnosis and intervention and demonstrate the substantial impact of EGPA on both patients and healthcare systems in Europe, underscoring the necessity for enhanced diagnosis and management strategies, particularly for patients in the vasculitic phase. Additionally, biologic use was linked to OCS-sparing effects and improved disease control. These findings highlight the need for further research to increase awareness of the diverse clinical needs of patients with EGPA according to their disease phase and to characterise the real-world impact of biologic treatment on these patients.

## Data Availability

For requests for access to anonymised subject level data, please contact the corresponding author.

## Supplementary material

**Please note:** supplementary material is not edited by the Editorial Office, and is uploaded as it has been supplied by the author. Supplementary material 00310-2025.SUPPLEMENT

## References

[1] Emmi G, Bettiol A, Gelain E, et al. Evidence-based guideline for the diagnosis and management of eosinophilic granulomatosis with polyangiitis. Nat Rev Rheumatol 2023; 19: 378–393. doi:10.1038/s41584-023-00958-w 37161084

[2] Grayson PC, Ponte C, Suppiah R, et al. 2022 American College of Rheumatology/European Alliance of Associations for Rheumatology classification criteria for eosinophilic granulomatosis with polyangiitis. Ann Rheum Dis 2022; 81: 309–314. doi:10.1136/annrheumdis-2021-221794 35110334

[3] Comarmond C, Pagnoux C, Khellaf M, et al. Eosinophilic granulomatosis with polyangiitis (Churg-Strauss): clinical characteristics and long-term followup of the 383 patients enrolled in the French Vasculitis Study Group cohort. Arthritis Rheum 2013; 65: 270–281. doi:10.1002/art.37721 23044708

[4] Fagni F, Bello F, Emmi G. Eosinophilic granulomatosis with polyangiitis: dissecting the pathophysiology. Front Med (Lausanne) 2021; 8: 627776. doi:10.3389/fmed.2021.627776 33718405 PMC7943470

[5] Greco A, Rizzo MI, De Virgilio A, et al. Churg-Strauss syndrome. Autoimmun Rev 2015; 14: 341–348. doi:10.1016/j.autrev.2014.12.004 25500434

[6] Cottin V, Bel E, Bottero P, et al. Respiratory manifestations of eosinophilic granulomatosis with polyangiitis (Churg-Strauss). Eur Respir J 2016; 48: 1429–1441. doi:10.1183/13993003.00097-2016 27587545

[7] Berti A, Boukhlal S, Groh M, et al. Eosinophilic granulomatosis with polyangiitis: the multifaceted spectrum of clinical manifestations at different stages of the disease. Expert Rev Clin Immunol 2020; 16: 51–61. doi:10.1080/1744666X.2019.1697678 31762336

[8] Yılmaz İ, Tutar N, Şimşek Z, et al. Clinical and serological features of eosinophilic and vasculitic phases of eosinophilic granulomatosis with poliangiitis: a case series of 15 patients. Turk Thorac J 2017; 18: 72–77. doi:10.5152/TurkThoracJ.2017.16040 29404165 PMC5783085

[9] Chung SA, Langford CA, Maz M, et al. 2021 American College of Rheumatology/Vasculitis Foundation guideline for the management of antineutrophil cytoplasmic antibody-associated vasculitis. Arthritis Rheumatol 2021; 73: 1366–1383. doi:10.1002/art.41773 34235894 PMC12327957

[10] Hellmich B, Sanchez-Alamo B, Schirmer JH, et al. EULAR recommendations for the management of ANCA-associated vasculitis: 2022 update. Ann Rheum Dis 2024; 83: 30–47. doi:10.1136/ard-2022-223764 36927642

[11] Lefebvre P, Duh MS, Lafeuille MH, et al. Burden of systemic glucocorticoid-related complications in severe asthma. Curr Med Res Opin 2017; 33: 57–65. doi:10.1080/03007995.2016.1233101 27627132

[12] Robson J, Doll H, Suppiah R, et al. Glucocorticoid treatment and damage in the anti-neutrophil cytoplasm antibody-associated vasculitides: long-term data from the European Vasculitis Study Group trials. Rheumatology (Oxford) 2015; 54: 471–481. doi:10.1093/rheumatology/keu366 25205825

[13] Volmer T, Effenberger T, Trautner C, et al. Consequences of long-term oral corticosteroid therapy and its side-effects in severe asthma in adults: a focused review of the impact data in the literature. Eur Respir J 2018; 52: 1800703. doi:10.1183/13993003.00703-2018 30190274

[14] Dalal AA, Duh MS, Gozalo L, et al. Dose-response relationship between long-term systemic corticosteroid use and related complications in patients with severe asthma. J Manag Care Spec Pharm 2016; 22: 833–847. 27348284 10.18553/jmcp.2016.22.7.833 PMC10397753

[15] Caminati M, Maule M, Bello F, et al. Biologics for eosinophilic granulomatosis with polyangiitis. Curr Opin Allergy Clin Immunol 2023; 23: 36–43. doi:10.1097/ACI.0000000000000875 36413432

[16] Wechsler ME, Akuthota P, Jayne D, et al. Mepolizumab reduced steroid burden for patients with eosinophilic granulomatosis with polyangiitis with and without a vasculitic phenotype. CHEST 2023; 164: A46–A49. doi:10.1016/j.chest.2023.07.089 PMC10349249 37312233

[17] Wechsler ME, Nair P, Terrier B, et al. Benralizumab versus mepolizumab for eosinophilic granulomatosis with polyangiitis. N Engl J Med 2024; 390: 911–921. doi:10.1056/NEJMoa2311155 38393328

[18] Jayne DRW, Terrier B, Hellmich B, et al. Mepolizumab has clinical benefits including oral corticosteroid sparing irrespective of baseline EGPA characteristics. ERJ Open Res 2024; 10: 00509-02023. doi:10.1183/23120541.00509-2023 38196889 PMC10772899

[19] Wechsler ME, Akuthota P, Jayne D, et al. Mepolizumab or placebo for eosinophilic granulomatosis with polyangiitis. N Engl J Med 2017; 376: 1921–1932. doi:10.1056/NEJMoa1702079 28514601 PMC5548295

[20] Maspero J, Adir Y, Al-Ahmad M, et al. Type 2 inflammation in asthma and other airway diseases. ERJ Open Res 2022; 8: 00576-02021. doi:10.1183/23120541.00576-2021 35923421 PMC9339769

[21] Nanzer AM, Dhariwal J, Kavanagh J, et al. Steroid-sparing effects of benralizumab in patients with eosinophilic granulomatosis with polyangiitis. ERJ Open Res 2020; 6: 00451-2020. doi:10.1183/23120541.00451-2020 33263051 PMC7682702

[22] Mohammad AJ, Hot A, Arndt F, et al. Rituximab for the treatment of eosinophilic granulomatosis with polyangiitis (Churg–Strauss). Ann Rheum Dis 2016; 75: 396–401. doi:10.1136/annrheumdis-2014-206095 25467294

[23] Akiyama M, Kaneko Y, Takeuchi T. Rituximab for the treatment of eosinophilic granulomatosis with polyangiitis: a systematic literature review. Autoimmun Rev 2021; 20: 102737. doi:10.1016/j.autrev.2020.102737 33340770

[24] Molina B, Padoan R, Urban ML, et al. Dupilumab for relapsing or refractory sinonasal and/or asthma manifestations in eosinophilic granulomatosis with polyangiitis: a European retrospective study. Ann Rheum Dis 2023; 82: 1587–1593. doi:10.1136/ard-2023-224756. 37734881

[25] Bettiol A, Urban ML, Padoan R, et al. Benralizumab for eosinophilic granulomatosis with polyangiitis: a retrospective, multicentre, cohort study. Lancet Rheumatol 2023; 5: e707–e715. doi:10.1016/S2665-9913(23)00243-6 38251561

[26] FDA. Mepolizumab (Nucala) prescribing information. Date last accessed: 13 May 2024. https://www.accessdata.fda.gov/drugsatfda_docs/label/2025/125526s007lbl.pdf

[27] EMA. Mepolizumab (Nucala) prescribing information. Date last accessed: 13 May 2023. www.ema.europa.eu/en/documents/product-information/nucala-epar-product-information_en.pdf

[28] EMA. Fasenra (benralizumab) prescribing information. Date last accessed: 8 May 2025. www.ema.europa.eu/en/documents/product-information/fasenra-epar-product-information_en.pdf

[29] FDA. Fasenra (benralizumab) prescribing information. Date last accessed: 8 May 2025. www.accessdata.fda.gov/drugsatfda_docs/label/2024/761070s021lbl.pdf

[30] Bell CF, Ajmera M, Meyers J. Retrospective analysis of the burden of illness of eosinophilic granulomatosis with polyangiitis (EGPA) versus asthma in commercially insured US patients. Cureus 2023; 15: e42241. doi:10.7759/cureus.42241 37605658 PMC10440019

[31] Bell CF, Blauer-Peterson C, Mao J. Burden of illness and costs associated with eosinophilic granulomatosis with polyangiitis: evidence from a managed care database in the United States. J Manag Care Spec Pharm 2021; 27: 1249–1259. doi:10.18553/jmcp.2021.21002 34165321 PMC10394225

[32] Jakes RW, Kwon N, Nordstrom B, et al. Burden of illness associated with eosinophilic granulomatosis with polyangiitis: a systematic literature review and meta-analysis. Clin Rheumatol 2021; 40: 4829–4836. doi:10.1007/s10067-021-05783-8 34159493 PMC8599408

[33] Hwee J, Harper L, Fu Q, et al. Prevalence, incidence and healthcare burden of eosinophilic granulomatosis with polyangiitis in the UK. ERJ Open Res 2024; 10: 00430-02023. doi:10.1183/23120541.00430-2023 38746859 PMC11089387

[34] Jakes RW, Kwon N, Huynh L, et al. Burden of eosinophilic granulomatosis with polyangiitis in Europe. ERJ Open Res 2024; 10: 00912-02023. doi:10.1183/23120541.00912-2023 39104949 PMC11299011

[35] Durel CA, Berthiller J, Caboni S, et al. Long-term followup of a multicenter cohort of 101 patients with eosinophilic granulomatosis with polyangiitis (Churg-Strauss). Arthritis Care Res (Hoboken) 2016; 68: 374–387. doi:10.1002/acr.22686 26315340

[36] Matucci A, Vivarelli E, Perlato M, et al. EGPA phenotyping: not only ANCA, but also eosinophils. Biomedicines 2023; 11: 776. doi:10.3390/biomedicines11030776 36979755 PMC10045549

[37] Terrier B, Jayne DRW, Hellmich B, et al. Clinical benefit of mepolizumab in eosinophilic granulomatosis with polyangiitis for patients with and without a vasculitic phenotype. ACR Open Rheumatol 2023; 5: 354–363. doi:10.1002/acr2.11571 37312233 PMC10349249

[38] Ishii T, Kunishige H, Kobayashi T, et al. Real-world safety and effectiveness of mepolizumab for patients with eosinophilic granulomatosis with polyangiitis in Japan: a 48-week interim analysis of the MARS study. Mod Rheumatol 2023; 34: 978–987. doi:10.1093/mr/road109 38100679

[39] Mathur SK, Silver J, MacKnight SD, et al. Real-world mepolizumab treatment in eosinophilic granulomatosis with polyangiitis reduces disease burden in the United States. Ann Allergy Asthma Immunol 2025; 134: 341–350 e342. doi:10.1016/j.anai.2024.11.004 39549986

[40] EMA. Xolair (omalizumab) prescribing information. Date last accessed: 8 May 2025. www.ema.europa.eu/en/documents/product-information/xolair-epar-product-information_en.pdf

[41] EMA. Cinqaero (reslizumab) prescribing information. Date last accessed: 8 May 2025. www.ema.europa.eu/en/documents/product-information/cinqaero-epar-product-information_en.pdf

[42] EMA. Dupixent (dupilumab) prescribing information. Date last accessed: 8 May 2025. www.ema.europa.eu/en/documents/product-information/dupixent-epar-product-information_en.pdf

[43] Steinfeld J, Bradford ES, Brown J, et al. Evaluation of clinical benefit from treatment with mepolizumab for patients with eosinophilic granulomatosis with polyangiitis. J Allergy Clin Immunol 2019; 143: 2170–2177. doi:10.1016/j.jaci.2018.11.041 30578883 PMC7254609

[44] Canzian A, Venhoff N, Urban ML, et al. Use of biologics to treat relapsing and/or refractory eosinophilic granulomatosis with polyangiitis: data from a European collaborative study. Arthritis Rheumatol 2021; 73: 498–503. doi:10.1002/art.41534 33001543

[45] Terrier B, Pugnet G, de Moreuil C, et al. Rituximab versus conventional therapeutic strategy for remission induction in eosinophilic granulomatosis with polyangiitis: a double-blind, randomized, controlled trial. Ann Intern Med 2025; 178: 1249–1257. doi:10.7326/ANNALS-24-03947 40720835

[46] Dutertre M, Pugnet G, De Moreuil C, et al. Long-term efficacy of remission-induction regimens for eosinophilic granulomatosis with polyangiitis. Arthritis Rheumatol 2023; 75: 0854.

[47] Yates M, Watts RA, Bajema IM, et al. EULAR/ERA-EDTA recommendations for the management of ANCA-associated vasculitis. Ann Rheum Dis 2016; 75: 1583–1594. doi:10.1136/annrheumdis-2016-209133 27338776

