# Supplementary material for: Burden of eosinophilic granulomatosis with polyangiitis by disease phase and steroid-sparing effects of biologics: a real-world retrospective study in Europe
Source: ERJ Open Res. 2026 Mar 2;12(2):00310-2025. doi: 10.1183/23120541.00310-2025 (PMC12951306; doi:10.1183/23120541.00310-2025)
Supplement: Supplementary file 1 [file 00310-2025.SUPPLEMENT.pdf]

Supplementary material

***Supplementary Table S1**: Additional patient demographics and clinical characteristics across EGPA disease phases*

Patient demographics and disease characteristics	Overall[a] (N=407)	Prodromal (N=36)	Eosinophilic (N=220)	Vasculitic (N=125)
**Demographic characteristics**				
**Age on index date, years**				
Median (IQR)	45.5 (33.6, 54.5)	42.6 (35.0, 54.2)	45.9 (33.6, 53.4)	44.5 (32.8, 54.8)
Paediatric (age 12–17), n (%)	21 (5.2)	2 (5.6)	13 (5.9)	6 (4.8)
Adult (age ≥18), n (%)	386 (94.8)	34 (94.4)	207 (94.1)	119 (95.2)
**Smoking status[b], n (%)**				
Never smoked	193 (47.4)	11 (30.6)	112 (50.9)	60 (48.0)
Former smoker	155 (38.1)	19 (52.8)	80 (36.4)	43 (34.4)
Current smoker	50 (12.3)	4 (11.1)	23 (10.5)	20 (16.0)
Unknown	9 (2.2)	2 (5.6)	5 (2.3)	2 (1.6)
**Year of diagnosis, n (%)**				
2014 or before	48 (11.8)	4 (11.1)	25 (11.4)	13 (10.4)
2015	21 (5.2)	1 (2.8)	10 (4.5)	10 (8.0)
2016	33 (8.1)	2 (5.6)	16 (7.3)	14 (11.2)
2017	37 (9.1)	3 (8.3)	21 (9.5)	11 (8.8)
2018	84 (20.6)	9 (25.0)	46 (20.9)	24 (19.2)
2019	184 (45.2)	17 (47.2)	102 (46.4)	53 (42.4)
**Disease duration (years from diagnosis date), years**				
Median (IQR)	2.5 (1.8, 4.1)	2.4 (1.8, 3.8)	2.4 (1.8, 3.9)	2.8 (1.9, 4.4)
n (%)				
<1 year	4 (1.0)	1 (2.8)	2 (0.9)	1 (0.8)

1–3 years	297 (73.0)	28 (77.8)	165 (75.0)	85 (68.0)
4–6 years	69 (17.0)	3 (8.3)	33 (15.0)	29 (23.2)
7–10 years	22 (5.4)	2 (5.6)	12 (5.5)	6 (4.8)
≥11 years	15 (3.7)	2 (5.6)	8 (3.6)	4 (3.2)
**Index year, n (%)**				
2015	43 (10.6)	2 (5.6)	25 (11.4)	11 (8.8)
2016	40 (9.8)	3 (8.3)	19 (8.6)	16 (12.8)
2017	39 (9.6)	3 (8.3)	19 (8.6)	16 (12.8)
2018	73 (17.9)	9 (25.0)	38 (17.3)	21 (16.8)
2019	212 (52.1)	19 (52.8)	119 (54.1)	61 (48.8)
**Duration between diagnosis date and index date, years**				
Median (IQR)	0.0 (0.0, 0.3)	0.0 (0.0, 0.3)	0.1 (0.0, 0.3)	0.0 (0.0, 0.2)
Patients with diagnosis date and index date on the same day, n (%)	162 (39.8)	17 (47.2)	77 (35.0)	60 (48.0)
**Length of follow-up from index date (years from index date), years**				
Mean (SD)	2.7 (1.4)	2.6 (1.4)	2.7 (1.5)	2.8 (1.4)
**Disease characteristics**				
**Diagnostic criteria[c], n (%)**				
ACR	273 (67.1)	19 (52.8)	151 (68.6)	88 (70.4)
CHCC	109 (26.8)	12 (33.3)	55 (25.0)	38 (30.4)
Other[d]	20 (4.9)	5 (13.9)	7 (3.2)	7 (5.6)
Unknown	41 (10.1)	2 (5.6)	25 (11.4)	7 (5.6)
**Diagnostic assessments[c], n (%)**				
Blood eosinophilia	381 (93.6)	31 (86.1)	211 (95.9)	115 (92.0)
Blood tests to screen for	372 (91.4)	27 (75.0)	203 (92.3)	119 (95.2)

autoimmunity (e.g. ANCA)				
Imaging scans of affected organs	338 (83.0)	28 (77.8)	182 (82.7)	111 (88.8)
Biopsy to detect extravascular eosinophils	203 (49.9)	17 (47.2)	100 (45.5)	75 (60.0)
Test to detect neuropathy	157 (38.6)	13 (36.1)	74 (33.6)	66 (52.8)
Other test of affected organs[e]	19 (4.7)	1 (2.8)	7 (3.2)	11 (8.8)
Unknown	3 (0.7)	0 (0.0)	1 (0.5)	0 (0.0)
**Number of diagnostic assessments[c], n (%)**				
1–3	98 (24.1)	11 (30.6)	61 (27.7)	16 (12.8)
4–6	189 (46.4)	13 (36.1)	99 (45.0)	65 (52.0)
7–12	117 (28.7)	12 (33.3)	59 (26.8)	44 (35.2)
**Asthma airway reversibility[f], n (%)**	**N=299**	**N=21**	**N=164**	**N=95**
Yes	264 (88.3)	21 (100.0)	149 (90.9)	78 (82.1)
No	26 (8.7)	0 (0.0)	11 (6.7)	13 (13.7)
Unknown	9 (3.0)	0 (0.0)	4 (2.4)	4 (4.2)
**Patients who had asthma diagnosis before EGPA diagnosis[g], n (%)**	160 (78.0)	10 (71.4)	84 (78.5)	60 (76.9)
Time from asthma diagnosis to EGPA diagnosis, years				
Median (IQR)	1.8 (0.2, 5.6)	3.0 (0.3, 8.9)	1.5 (0.1, 4.3)	2.9 (0.1, 6.1)

[a]Overall (N=407) included patients with eosinophilic (N=220), vasculitic (N=125), prodromal (N=36) and unknown (N=26) disease phases; [b]smoking status was assessed at the time of chart extraction; [c]diagnostic criteria and diagnostic assessments are not mutually exclusive categories. All the categories of diagnostic assessment were counted for this statistic. For example, if a physician indicated that a patient had an imaging scan of affected organs, and then further specified that the patient had an abdominal CT and a chest radiograph, this would count as two diagnostic assessments; [d]other diagnostic criteria included EULAR (8 patients), BTS (5), SPLF (2), PRES (1). Five charts also

indicated diagnostic criteria guided by other clinical assessments (e.g. BSR, blood tests, biopsies) and local guidelines (5); [e] other diagnostic assessments included electromyography (2), renal biopsy (2), rhinoscopy (2), biopsy of bronchopulmonary (1), bronchoscopy (1), capillary microscopy (1), cardiology (1), endoscopy (1) and other tests associated with eye, kidney, skin, lung and urinary systems (7); [f] asthma airway reversibility was assessed for 299 patients who had asthma between EGPA diagnosis and EOF; [g] 45 patients had an asthma diagnosis after EGPA diagnosis. 94 patients who had asthma between EGPA diagnosis and EOF did not have a reported asthma diagnosis date.

ACR, American College of Rheumatology; ANCA, antineutrophil cytoplasmic antibodies; BSR, British Society of Rheumatology; BTS, British Thoracic Society; CHCC, Chapel Hill Consensus Conference; CT, computed tomography; ENT, ear, nose and throat; EGPA, eosinophilic granulomatosis with polyangiitis; EOF, end of follow-up; EULAR, European Alliance of Associations for Rheumatology; IQR, interquartile range; PRES, posterior reversible encephalopathy syndrome; SPLF, Société de Pneumologie de Langue Francaise.

Supplementary Table S2. *Baseline patient demographics and disease characteristics stratified by physician speciality*

Patient demographics and disease characteristics	Overall (N=407)	Allergy (N=60)	Immunology (N=28)	Rheumatology (N=165)	Pulmonology (N=154)
**Patient country, n (%)**					
France	81 (19.9)	2 (3.3)	3 (10.7)	15 (9.1)	61 (39.6)
Germany	80 (19.7)	12 (20.0)	0 (0.0)	45 (27.3)	23 (14.9)
Italy	80 (19.7)	8 (13.3)	21 (75.0)	27 (16.4)	24 (15.6)
Spain	85 (20.9)	32 (53.3)	4 (14.3)	30 (18.2)	19 (12.3)
UK	81 (19.9)	6 (10.0)	0 (0.0)	48 (29.1)	27 (17.5)
**Age at EGPA diagnosis, years**					
Median (IQR)	44.5 (32.8, 53.7)	45.2 (30.9, 54.4)	30.9 (19.8, 44.5)	43.5 (31.4, 53.1)	46.6 (35.2, 55.0)
≥18 years of age, n (%)	383 (94.1)	53 (88.3)	23 (82.1)	154 (93.3)	153 (99.4)
**Male, n (%)**	231 (56.8)	32 (53.3)	17 (60.7)	96 (58.2)	86 (55.8)
**EGPA diagnosis, n (%)**					
Before study (≤2014)	48 (11.8)	3 (5.0)	4 (14.3)	23 (13.9)	18 (11.7)
During study (2015–2019)	359 (88.2)	57(95.0)	24 (85.7)	142 (86.1)	136 (88.3)
**Disease duration (between EGPA diagnosis and EOF), median (IQR)**	2.5 (1.8, 4.1)	2.6 (1.9, 4.1)	2.6 (1.9, 4.6)	2.6 (1.8, 4.1)	2.3 (1.7, 4.1)
**Disease phase[a] n (%)**					
Prodromal	36 (8.8)	5 (8.3)	5 (17.9)	12 (7.3)	14 (9.1)
Eosinophilic	220 (54.1)	45 (75.0)	13 (46.4)	73 (44.2)	89 (57.8)
Vasculitic	125 (30.7)	6 (10.0)	10 (35.7)	70 (42.4)	39 (25.3)
Unknown	26 (6.4)	4 (6.7)	0 (0.0)	10 (6.1)	12 (7.8)
**Comorbidities, n (%)**					
Vasculitis	197 (48.4)	24 (40.0)	14 (50.0)	89 (53.9)	70 (45.5)
Hypertension[b]	163 (40.0)	20 (33.3)	10 (35.7)	73 (44.2)	60 (39.0)
Anxiety or depression	140 (34.4)	22 (36.7)	11 (39.3)	43 (26.1)	64 (41.6)
Lower respiratory disease(s)[c]	77 (18.9)	10 (16.7)	7 (25.0)	32 (19.4)	28 (18.2)
Osteoporosis[b]	72 (17.7)	11 (18.3)	4 (14.3)	33 (20.0)	24 (15.6)
Glomerulonephritis	69 (17.0)	6 (10.0)	1 (3.6)	32 (19.4)	30 (19.5)

Obesity	68 (16.7)	18 (30.0)	4 (14.3)	28 (17.0)	18 (11.7)
Diabetes[b]	35 (8.6)	8 (13.3)	1 (3.6)	13 (7.9)	13 (8.4)
Rheumatoid arthritis	20 (4.9)	6 (10.0)	1 (3.6)	4 (2.4)	9 (5.8)
Liver disease	11 (2.7)	3 (5.0)	0 (0.0)	7 (4.2)	1 (0.6)
Other	22 (5.4)	3 (5.0)	4 (14.3)	8 (4.8)	7 (4.5)
Cancer (any)[d]	8 (2.0)	1 (1.7)	1 (3.6)	3 (1.8)	3 (1.9)
**Blood eosinophil count**					
n (%)	364 (89.4)	53 (88.3)	22 (78.6)	146 (88.5)	143 (92.9)
Median (IQR), cell/µL	1,500.0 (600.0, 3,300.0)	2,450.0 (756.0, 5,646.0)	1,700.0 (900.0, 3,500.0)	1,800.0 (940.0, 3,700.0)	1,150.0 (459.0, 2,000.0)
**Proportion of patients with asthma, n (%)**	299 (73.5)	43 (71.7)	19 (67.9)	105 (63.6)	132 (85.7)
**Time from asthma diagnosis to EGPA diagnosis, median (IQR)[e], years**	1.8 (0.2, 5.6)	2.3 (0.0, 13.5)	3.0 (0.1, 4.4)	2.9 (1.0, 6.2)	1.0 (0.0, 4.5)
**Length of follow-up, mean (SD), years**	2.7 (1.4)	2.7 (1.6)	2.6 (1.4)	2.9 (1.4)	2.6 (1.4)

[a]Disease phase was assessed on or before index date; [b]Potentially steroid-related; [c]Other than asthma and COPD; [d]reported cancer types included: lung (3), breast (1), colon (1), leukaemia (1), rectum (1), skin (1); [e]among 154 patients who had asthma diagnosed before EGPA.

***Supplementary Table S3**: Detailed treatment patterns by EGPA disease phases at any time from diagnosis*

	**Overall (N=407)**	**Prodromal (N=36)**	**Eosinophilic (N=220)**	**Vasculitic (N=125)**
**Number of distinct EGPA therapies used[a]**				
Mean (SD)	3.9 (1.8)	3.8 (2.1)	3.7 (1.8)	4.4 (1.7)
**Number of distinct EGPA therapies used, categorical[a], n (%)**				
1–2	102 (25.1)	13 (36.1)	63 (28.6)	17 (13.6)
3–4	166 (40.8)	10 (27.8)	96 (43.6)	50 (40.0)
5–7	126 (31.0)	12 (33.3)	53 (24.1)	54 (43.2)
≥8	13 (3.2)	1 (2.8)	8 (3.6)	4 (3.2)
**Time from diagnosis to initiation of therapy, median (IQR), years**	0.0 (0.0, 0.1)	0.0 (0.0, 0.4)	0.0 (0.0, 0.1)	0.0 (0.0, 0.1)
**Time from diagnosis to initiation of biologics, median (IQR), years**	1.4 (0.5, 2.7)	1.5 (1.2, 2.3)	1.4 (0.6, 2.6)	1.3 (0.2, 3.1)
**EGPA therapies by treatment category**				
**Oral corticosteroids, n (%)**	**402 (98.8)**	**35 (97.2)**	**218 (99.1)**	**123 (98.4)**
Patients with a reported maximum daily dose for maintenance therapy, n (%)	314 (77.1)	29 (80.6)	171 (77.7)	97 (77.6)
Maximum daily dose across all oral corticosteroids[b], mg				
Mean (SD)	30.2 (19.8)	37.8 (19.5)	29.1 (19.6)	29.8 (19.7)
Median (IQR)	30.0 (10.0, 50.0)	40.0 (20.0, 60.0)	25.0 (10.0, 50.0)	30.0 (10.0, 50.0)
Prednisone or prednisolone, n (%)	349 (85.7)	25 (69.4)	187 (85.0)	112 (89.6)
Methylprednisolone, n (%)	98 (24.1)	12 (33.3)	54 (24.5)	29 (23.2)
Cortisone, n (%)	19 (4.7)	6 (16.7)	7 (3.2)	6 (4.8)
**Immunosuppressive and cytotoxic therapies[c], n (%)**	**260 (63.9)**	**26 (72.2)**	**112 (50.9)**	**103 (82.4)**
Azathioprine	110 (27.0)	7 (19.4)	50 (22.7)	45 (36.0)
Cyclophosphamide	78 (19.2)	9 (25.0)	31 (14.1)	33 (26.4)
Methotrexate	77 (18.9)	7 (19.4)	29 (13.2)	36 (28.8)
**Biologics, n (%)**	185 (45.5)	14 (38.9)	106 (48.2)	62 (49.6)
Mepolizumab	74 (18.2)	3 (8.3)	46 (20.9)	25 (20.0)
Rituximab	74 (18.2)	6 (16.7)	34 (15.5)	33 (26.4)

Benralizumab	26 (6.4)	1 (2.8)	20 (9.1)	5 (4.0)
Omalizumab	18 (4.4)	1 (2.8)	12 (5.5)	3 (2.4)
Reslizumab	16 (3.9)	3 (8.3)	12 (5.5)	1 (0.8)
Dupilumab	1 (0.2)	0 (0.0)	1 (0.5)	0 (0.0)
**Other treatments for the control of EGPA-related clinical manifestations[c], n (%)**	**302 (74.2)**	**22 (61.1)**	**161 (73.2)**	**101 (80.8)**
Budesonide/formoterol	160 (39.3)	12 (33.3)	90 (40.9)	46 (36.8)
Albuterol	119 (29.2)	9 (25.0)	60 (27.3)	39 (31.2)
Analgesics	96 (23.6)	7 (19.4)	44 (20.0)	41 (32.8)
Montelukast	62 (15.2)	8 (22.2)	31 (14.1)	19 (15.2)
Ramipril	55 (13.5)	5 (13.9)	21 (9.5)	26 (20.8)
Tiotropium	45 (11.1)	2 (5.6)	29 (13.2)	12 (9.6)
**Other treatments used related to the complications and adverse effects of immunosuppressive medications[d], n (%)**	**238 (58.5)**	**22 (61.1)**	**121 (55.0)**	**84 (67.2)**
Treatments for the improvement of bone mineral density[e]	163 (40.0)	10 (27.8)	81 (36.8)	64 (51.2)
Folic acid supplements	88 (21.6)	8 (22.2)	41 (18.6)	38 (30.4)
Treatment for infections in relation to EGPA therapies[f]	55 (13.5)	8 (22.2)	21 (9.5)	25 (20.0)
Treatments for gastrointestinal toxicity	40 (9.8)	0 (0.0)	20 (9.1)	18 (14.4)
Thyroid hormone replacement treatments	22 (5.4)	0 (0.0)	11 (5.0	10 (8.0)
Treatments for recovering cytopenia	21 (5.2)	2 (5.6)	12 (5.5)	5 (4.0)
Treatments for haemorrhagic cystitis	18 (4.4)	6 (16.7)	8 (3.6)	4 (3.2)
Insulin	10 (2.5)	1 (2.8)	5 (2.3)	4 (3.2)
Treatments for hepatotoxicity	7 (1.7)	1 (2.8)	5 (2.3)	1 (0.8)

[a]Receipt of one or multiple oral corticosteroid drugs was counted as a single therapy; [b]for patients who received ≥1 prescriptions for a drug, the maximum dosage was reported as the maximum dosage across all prescriptions. Patients with reported maximum daily dose for maintenance therapy over 60 mg were removed from the summary of maximum daily dose statistics, as these values seemed most likely to reflect dosing for burst treatment episodes instead of for maintenance therapy; [c]only treatments used by >10% of overall patients are listed, treatments used by <10% of overall patients included doxazosin, ipratropium bromide, levalbuterol, theophylline, valsartan and zafirlukast; [d]treatment use in this category was assessed between index date and EOF; [e]examples of treatments for the improvement of bone mineral density include alendronate, risedronate sodium, ibandronate and zoledronic acid; [f]examples of treatments for infection in relation to EGPA therapies include antitubercular agents, acyclovir, valacyclovir and fluconazole.

EGPA, eosinophilic granulomatosis with polyangiitis; EOF, end of follow-up; IQR, interquartile range; SD, standard deviation.

***Supplementary Table S4**: Specific clinical manifestations across EGPA disease phases*

**Clinical manifestations[a]**	**Overall (N=407)**	**Prodromal (N=36)**	**Eosinophilic (N=220)**	**Vasculitic (N=125)**
**Number of distinct clinical manifestations[a]**				
Median (IQR)	3.0 (1.0, 6.0)	3.0 (1.5, 6.0)	3.0 (1.0, 5.0)	4.0 (2.0, 7.0)
**Number of distinct clinical manifestations, categorical[a], n (%)**				
0	63 (15.5)	3 (8.3)	38 (17.3)	16 (12.8)
1–2	107 (26.3)	14 (38.9)	60 (27.3)	28 (22.4)
3–5	131 (32.2)	9 (25.0)	74 (33.6)	35 (28.0)
6–8	61 (15.0)	6 (16.7)	28 (12.7)	26 (20.8)
9–12	30 (7.4)	2 (5.6)	12 (5.5)	15 (12.0)
≥13	15 (3.7)	2 (5.6)	8 (3.6)	5 (4.0)
**Clinical manifestations by organ involvement[a]**				
**Lung, n (%)**	**227 (55.8)**	**22 (61.1)**	**123 (55.9)**	**69 (55.2)**
Shortness of breath	151 (37.1)	15 (41.7)	74 (33.6)	53 (42.4)
Lung infiltrates	105 (25.8)	10 (27.8)	52 (23.6)	37 (29.6)
Severe asthma	101 (24.8)	6 (16.7)	64 (29.1)	29 (23.2)
Pleural effusion	21 (5.2)	2 (5.6)	12 (5.5)	6 (4.8)
Alveolar haemorrhage	15 (3.7)	2 (5.6)	7 (3.2)	6 (4.8)
**Ear, nose and throat, n (%)**	**217 (53.3)**	**22 (61.1)**	**113 (51.4)**	**70 (56.0)**
Allergic rhinitis	140 (34.4)	11 (30.6)	77 (35.0)	42 (33.6)
Paranasal sinusitis	90 (22.1)	11 (30.6)	45 (20.5)	29 (23.2)
Nasal polyposis	89 (21.9)	10 (27.8)	45 (20.5)	30 (24.0)
Otitis media	14 (3.4)	5 (13.9)	7 (3.2)	2 (1.6)
**Constitutional, n (%)**	**198 (48.6)**	**19 (52.8)**	**94 (42.7)**	**74 (59.2)**
Fatigue	174 (42.8)	17 (47.2)	78 (35.5)	70 (56.0)

Myalgia/arthralgia	111 (27.3)	7 (19.4)	51 (23.2)	44 (35.2)
**Skin, n (%)**	**170 (41.8)**	**17 (47.2)**	**80 (36.4)**	**65 (52.0)**
Itch	78 (19.2)	9 (25.0)	41 (18.6)	27 (21.6)
Urticaria	74 (18.2)	8 (22.2)	39 (17.7)	27 (21.6)
Purpura	65 (16.0)	7 (19.4)	24 (10.9)	27 (21.6)
Ulcers	27 (6.6)	1 (2.8)	14 (6.4)	12 (9.6)
**Gastrointestinal, n (%)**	**79 (19.4)**	**9 (25.0)**	**36 (16.4)**	**33 (26.4)**
Abdominal pain	43 (10.6)	5 (13.9)	21 (9.5)	16 (12.8)
Diarrhoea	31 (7.6)	4 (11.1)	14 (6.4)	13 (10.4)
Nausea/vomiting	25 (6.1)	0 (0.0)	13 (5.9)	11 (8.8)
Gastrointestinal bleeding	17 (4.2)	1 (2.8)	7 (3.2)	9 (7.2)
**Renal, n (%)**	**78 (19.2)**	**5 (13.9)**	**33 (15.0)**	**38 (30.4)**
Glomerulonephritis	43 (10.6)	1 (2.8)	20 (9.1)	21 (16.8)
Proteinuria	39 (9.6)	2 (5.6)	17 (7.7)	18 (14.4)
Haematuria	22 (5.4)	3 (8.3)	7 (3.2)	11 (8.8)
**Cardiovascular, n (%)**	**45 (11.1)**	**2 (5.6)**	**17 (7.7)**	**26 (20.8)**
Cardiac arrhythmia	18 (4.4)	1 (2.8)	10 (4.5)	7 (5.6)
Ischaemic heart disease	14 (3.4)	1 (2.8)	3 (1.4)	10 (8.0)
Cardiomyopathy	9 (2.2)	1 (2.8)	6 (2.7)	2 (1.6)
Peripheral vascular disease	9 (2.2)	0 (0.0)	4 (1.8)	5 (4.0)
Valvular disease	9 (2.2)	1 (2.8)	4 (1.8)	4 (3.2)
Pericarditis	8 (2.0)	0 (0.0)	4 (1.8)	4 (3.2)
Heart failure	7 (1.7)	1 (2.8)	5 (2.3)	1 (0.8)
**Neuropsychiatric, n (%)**	**75 (18.4)**	**8 (22.2)**	**37 (16.8)**	**29 (23.2)**
Peripheral neuropathy	51 (12.5)	5 (13.9)	28 (12.7)	17 (13.6)
Mononeuritis	22 (5.4)	3 (8.3)	10 (4.5)	9 (7.2)

Cranial nerve palsies or involvement	6 (1.5)	2 (5.6)	3 (1.4)	1 (0.8)
Psychosis	5 (1.2)	0 (0.0)	4 (1.8)	1 (0.8)
Stroke	5 (1.2)	0 (0.0)	4 (1.8)	1 (0.8)
**Biopsy confirmed eosinophilic vasculitis or eosinophilic inflammation[b], n (%)**	**42 (10.3)**	**4 (11.1)**	**19 (8.6)**	**19 (15.2)**

[a]Manifestations are reported from index date to EOF; [b]the biopsy sites were skin (n=20), lung (9), kidney (3), muscle (2), alveoli (1), EEII (1), intestine (1), peripheral nerve (1) and unknown (4).

EGPA, eosinophilic granulomatosis with polyangiitis; EEII, lower extremities; EOF, end of follow-up; IQR, interquartile range.

***Supplementary Table S5**: EGPA-related HCRU of patients across EGPA disease phase subgroups*

	**Overall (N=407)**	**Prodromal (N=36)**	**Eosinophilic (N=220)**	**Vasculitic (N=125)**
**EGPA-related visits[a]**				
**Hospitalizations, n (%)**	149 (36.6)	12 (33.3)	60 (27.3)	72 (57.6)
Number of visits, PPPY, mean (SD)	0.5 (1.3)	0.3 (0.6)	0.3 (0.7)	0.9 (2.0)
Average length of stay per hospitalization, days[b], mean (SD)	8.4 (6.6)	6.1 (4.4)	7.7 (7.4)	9.2 (6.3)
**ER visits, n (%)**	106 (26.0)	16 (44.4)	42 (19.1)	41 (32.8)
Number of visits, PPPY, mean (SD)	0.3 (1.0)	0.5 (0.8)	0.3 (1.0)	0.4 (0.8)
**Outpatient visits, n (%)**	345 (84.8)	27 (75.0)	185 (84.1)	109 (87.2)
Number of visits, PPPY, mean (SD)	5.9 (38.0)	3.1 (2.5)	6.5 (47.8)	6.3 (26.2)
Unscheduled outpatient visits, PPPY, mean (SD)	0.7 (0.9)	0.8 (0.9)	0.6 (0.8)	0.9 (1.1)
**Proportion of patients with any occurrence of tests[a] related to the complications and monitoring of adverse effects of using immunosuppressive medications, n (%)**				
EGPA-related medical procedure (e.g. plasma exchange)	52 (12.8)	7 (19.4)	29 (13.2)	16 (12.8)
Number PPPY, mean (SD)	1.7 (2.6)	1.0 (0.5)	2.1 (3.4)	1.2 (1.0)
Bone mineral density testing	118 (29.0)	12 (33.3)	55 (25.0)	47 (37.6)
Number PPPY, mean (SD)	0.7 (0.4)	1.0 (0.6)	0.7 (0.4)	0.8 (0.4)
Cataract removal	5 (1.2)	1 (2.8)	2 (0.9)	1 (0.8)
Number PPPY, mean (SD)	0.9 (0.9)	0.7 (0.0)	1.4 (1.4)	0.7 (0.0)
Imaging tests	116 (28.5)	15 (41.7)	56 (25.5)	43 (34.4)
Number PPPY, mean (SD)	1.4 (1.3)	1.1 (0.6)	1.5 (1.7)	1.5 (1.1)

[a]EGPA-related visits and tests were assessed between index date and EOF; [b]length of stay is calculated among patients with inpatient visits.

EGPA, eosinophilic granulomatosis with polyangiitis; EOF, end of follow-up; ER, emergency room; HCRU, healthcare resource utilization; PPPY, per-patient-per-year; SD, standard deviation.

***Supplementary Table S6**: Additional demographics and disease characteristics of patients with a history of EGPA receiving biologics*

Patient demographics and disease characteristics	Overall[a] (N=407)	Biologics exposed (n=185)
**Demographic characteristics**		
**Age category at EGPA diagnosis, years**		
Paediatric (age 12–17), n (%)	24 (5.9)	9 (4.9)
Adult (age ≥18), n (%)	383 (94.1)	176 (95.1)
**Age on index date, years**		
Median (IQR)	45.5 (33.6, 54.5)	44.8 (34.8, 53.1)
Paediatric (age 12–17), n (%)	21 (5.2)	7 (3.8)
Adult (age ≥18), n (%)	386 (94.8)	178 (96.2)
**Smoking status[b], n (%)**		
Never smoked	193 (47.4)	82 (44.3)
Former smoker	155 (38.1)	73 (39.5)
Current smoker	50 (12.3)	27 (14.6)
Unknown	9 (2.2)	3 (1.6)
**Year of diagnosis, n (%)**		
2014 or before	48 (11.8)	23 (12.4)
2015	21 (5.2)	10 (5.4)
2016	33 (8.1)	16 (8.6)
2017	37 (9.1)	20 (10.8)
2018	84 (20.6)	39 (21.1)
2019	184 (45.2)	77 (41.6)
**Disease duration (years from diagnosis date)**		
Median (IQR)	2.5 (1.8, 4.1)	2.6 (1.9, 4.4)
n (%)		
<1 year	4 (1.0)	3 (1.6)

1–3 years	297 (73.0)	132 (71.4)
4–6 years	69 (17.0)	32 (17.3)
7–10 years	22 (5.4)	13 (7.0)
≥11 years	15 (3.7)	5 (2.7)
**Index year, n (%)**		
2015	43 (10.6)	21 (11.4)
2016	40 (9.8)	18 (9.7)
2017	39 (9.6)	22 (11.9)
2018	73 (17.9)	33 (17.8)
2019	212 (52.1)	91 (49.2)
**Duration between diagnosis date and index date, years**		
Median (IQR)	0.0 (0.0, 0.3)	0.1 (0.0, 0.3)
Patients with diagnosis date and index date on the same day, n (%)	162 (39.8)	70 (37.8)
**Length of follow-up from index date, years**		
Mean (SD)	2.7 (1.4)	2.8 (1.5)
**Disease characteristics**		
**Diagnostic criteria[c], n (%)**		
ACR	273 (67.1)	121 (65.4)
CHCC	109 (26.8)	72 (38.9)
Other[d]	20 (4.9)	9 (4.9)
Unknown	41 (10.1)	7 (3.8)
**Diagnostic assessments[c], n (%)**		
Blood eosinophilia	381 (93.6)	176 (95.1)
Blood tests to screen for autoimmunity (e.g. ANCA)	372 (91.4)	167 (90.3)
Imaging scans of affected organs[e]	338 (83.0)	158 (85.4)
Biopsy to detect extravascular eosinophils	203 (49.9)	107 (57.8)
Test to detect neuropathy	157 (38.6)	85 (45.9)

Other test of affected organs[f]	19 (4.7)	11 (5.9)
Unknown	3 (0.7)	1 (0.5)
**Number of diagnostic assessments[c], n (%)**		
1–3	98 (24.1)	34 (18.4)
4–6	189 (46.4)	95 (51.4)
7–12	117 (28.7)	55 (29.7)
**Asthma airway reversibility[g], n (%)**	**N=299**	**N=147**
Yes	264 (88.3)	129 (87.8)
No	26 (8.7)	15 (10.2)
Unknown	9 (3.0)	3 (2.0)
**Patients who had asthma diagnosis before EGPA diagnosis[h] n (%)**	160 (78.0)	76 (67.9)
Time from asthma diagnosis to EGPA diagnosis, years		

[a]Overall (N=407) included patients who were exposed to biologics between EGPA diagnosis and EOF (N=185) and patients who were not exposed to biologics between EGPA diagnosis and EOF (N=222); [b]smoking status was assessed at the time of chart extraction; [c]diagnostic criteria and diagnostic assessments are not mutually exclusive categories. All the categories of diagnostic assessment were counted for this statistic. For example, if a physician indicated that a patient had an imaging scan of affected organs, and then further specified that the patient had an abdominal CT and a chest radiograph, this would count as two diagnostic assessments; [d]other diagnostic criteria included EULAR (8 patients), BTS (5), SPLF (2), PRES (1). Five charts also indicated diagnostic criteria guided by other clinical assessments (e.g. BSR, blood tests, biopsies) and local guidelines (5); [e]other imaging scans included PET (3) and scan associated with ENT (1); [f]other diagnostic assessments included electromyography (2), renal biopsy (2), rhinoscopy (2), biopsy of bronchopulmonary (1), bronchoscopy (1), capillary microscopy (1), cardiology (1), endoscopy (1) and other tests associated with eye, kidney, skin, lung and urinary systems (7); [g]asthma airway reversibility was assessed for 299 patients who had asthma between EGPA diagnosis and EOF; [h]45 patients had an asthma diagnosis after EGPA diagnosis. 94 patients who had asthma between EGPA diagnosis and EOF did not have a reported asthma diagnosis date.

ACR, American College of Rheumatology; ANCA, antineutrophil cytoplasmic antibodies; BSR, British Society of Rheumatology; BTS, British Thoracic Society; CHCC, Chapel Hill Consensus Conference; CT, computed tomography; ENT, ear, nose and throat; EGPA, eosinophilic granulomatosis with polyangiitis; EOF, end of follow-up; EULAR, European Alliance of Associations for Rheumatology; IQR, interquartile range; PET, positron emission tomography; PRES, posterior reversible encephalopathy syndrome; SD, standard deviation; SPLF, Société de Pneumologie de Langue Francaise.

Supplementary Table S7: *HCRU of patients with a history of EGPA, by biologics use*

**HCRU**	**Biologics-exposed subgroup (n=185)**
**Proportion of patients with any EGPA-related visits[a], n (%)**	
**Hospitalizations, n (%)**	91 (49.2)
Number of visits, PPPY, mean (SD)	0.6 (1.0)
Average length of stay per hospitalization (days)[b], mean (SD)	8.6 (7.4)
**ER visits, n (%)**	63 (34.1)
Number of visits, PPPY, mean (SD)	0.6 (1.3)
**Outpatient visits, n (%)**	163 (88.1)
Overall number of visits, PPPY, mean (SD)	7.8 (52.1)
Unscheduled outpatient visits, PPPY, mean (SD)	0.9 (1.0)
**Patients with any occurrence of tests related to the complications and monitoring of adverse effects of using immunosuppressive medications[a], n (%)**	
EGPA-related medical procedure (e.g. plasma exchange)	38 (20.5)
Number of occurrences PPPY, mean (SD)	1.9 (3.0)
Bone mineral density testing	66 (35.7)
Number of occurrences PPPY, mean (SD)	0.8 (0.4)
Cataract removal	2 (1.1)
Number of occurrences PPPY, mean (SD)	1.4 (1.4)
Imaging tests	64 (34.6)
Number of occurrences PPPY, mean (SD)	1.8 (1.7)

[a]EGPA-related visits and tests were assessed between index date and EOF. The number of hospitalizations, ER visits, outpatient visits, and various types of tests were annualized; [b]length of stay is calculated among patients with inpatient visits.

EGPA, eosinophilic granulomatosis with polyangiitis; EOF, end of follow-up; ER, emergency room; HCRU, healthcare resource utilization; PPPY, per-patient-per-year; SD, standard deviation.

Supplementary Figure S1. *A) Study design and B) subgroup analyses*

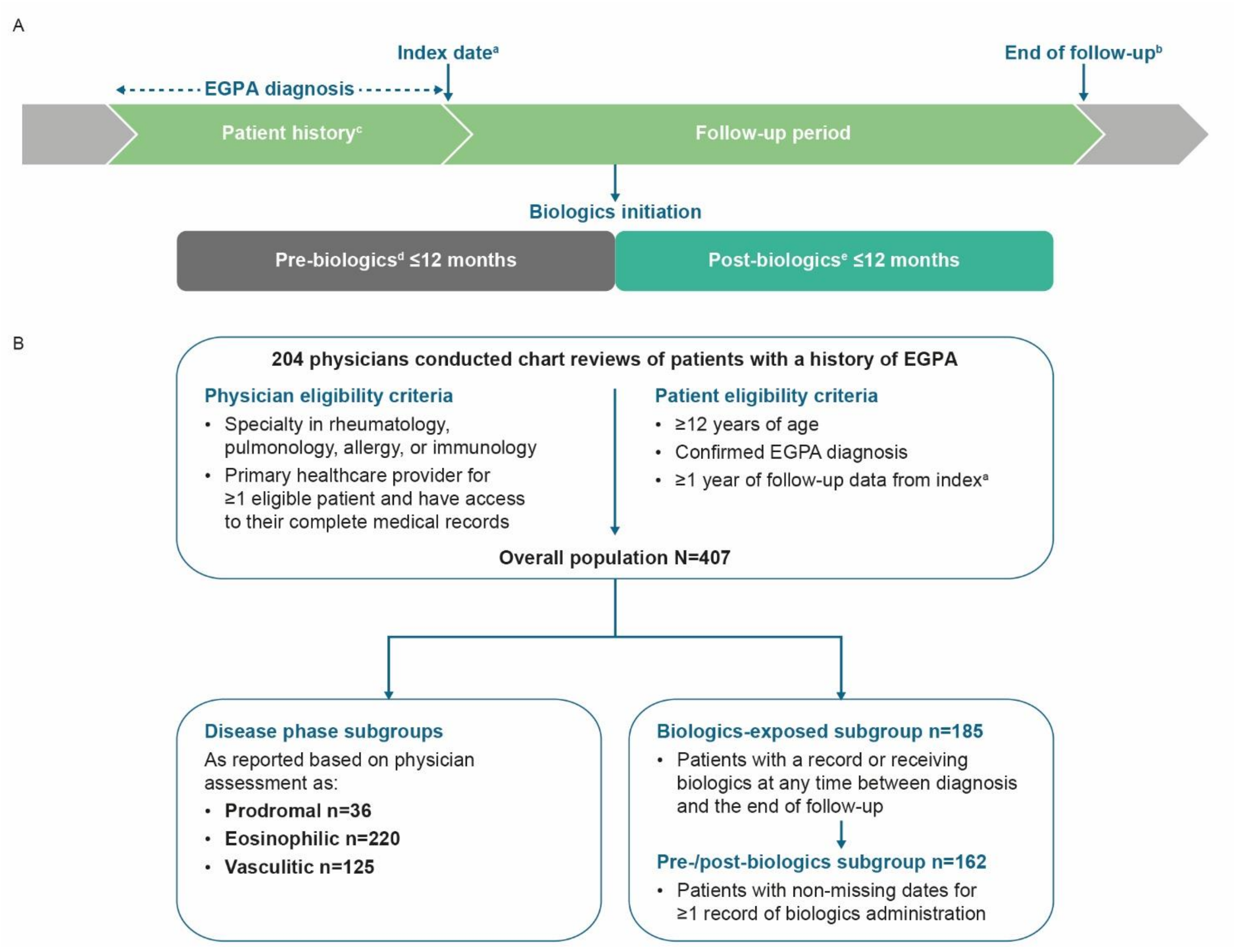

[a]Index date was the date of first physician visit, after or at EGPA diagnosis, between January 2015 and December 2019; [b]EOF was the earliest of death, loss to follow-up or date of chart abstraction; [c]a pre-index patient history period was developed to collect demographics and baseline clinical characteristics for patients diagnosed with EGPA before the index date. These data were also collected for patients diagnosed on the index date; [d]the pre-biologics period was defined as 12 months before and including the initiation of biologics. Only events and person-years after EGPA diagnosis were included; [e]the post-biologics period was defined as 12 months after the initiation of biologics, or until death or EOF.

EGPA, eosinophilic granulomatosis with polyangiitis; EOF, end of follow-up.

Supplementary Figure S2. *Real-world relapse-free survival across A) overall population, B) prodromal disease phase subgroup, C) eosinophilic disease phase subgroup, D) vasculitic disease phase subgroup*

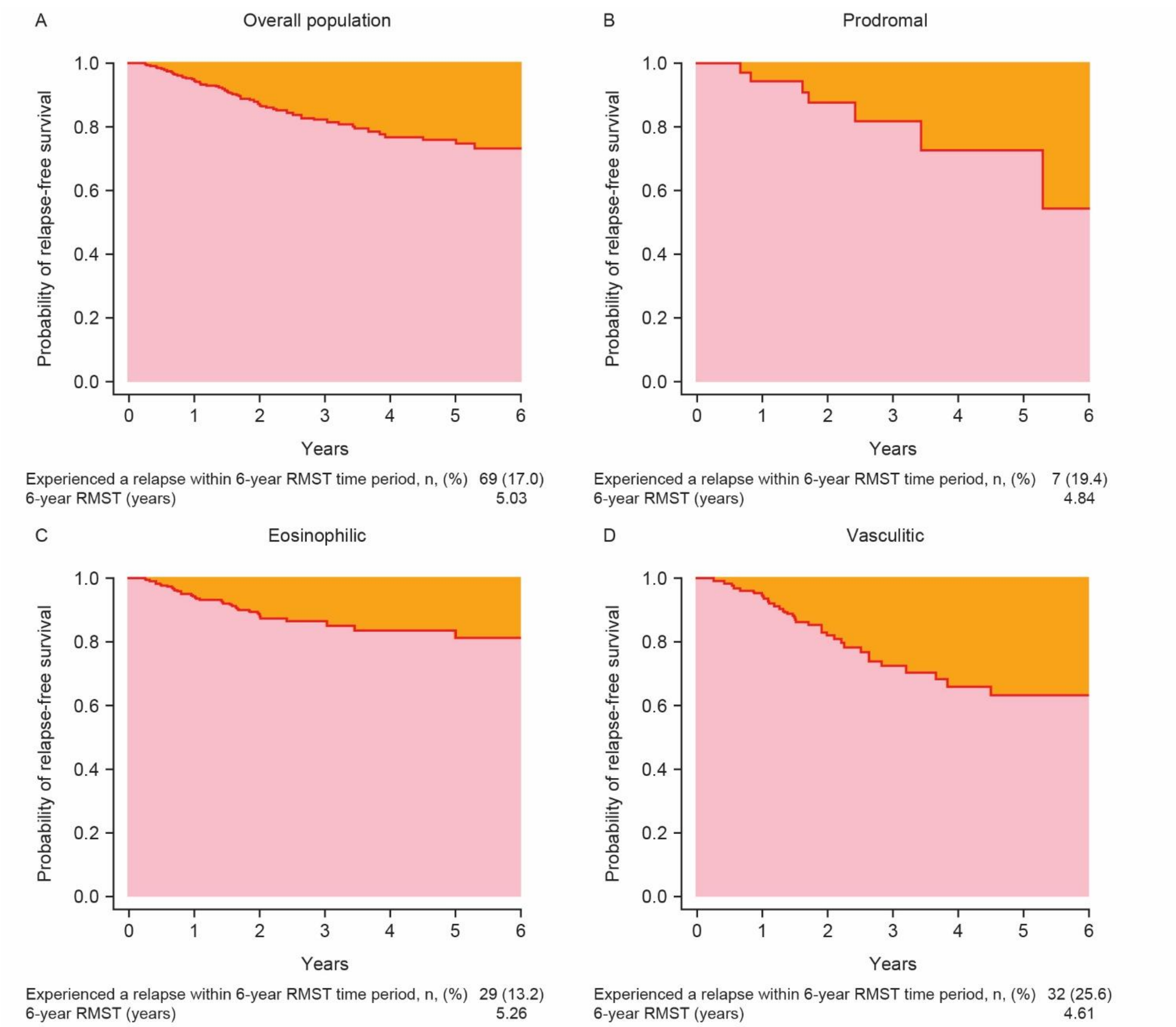

Relapse-free survival was calculated from the date of EGPA diagnosis to first relapse. Patients without record of relapse were censored at EOF or at 6 years after the diagnosis date, whichever came first. Patients with record of relapse were censored at 6 years after the diagnosis date if the duration between EGPA diagnosis and first relapse was over 6 years. Because the incidence of relapse was observed to be <50%, the median survival time could not be estimated, and RMST was reported instead. RMST is a measure of the average EFS time and is estimated by the area under the KM curve during a specified period of time.

EFS, event-free survival; EGPA, eosinophilic granulomatosis with polyangiitis; EOF, end of follow-up; KM, Kaplan–Meier; RMST, restricted mean survival time.

Supplementary Figure S3. *Real-world overall survival across A) overall population, B) prodromal disease phase subgroup, C) eosinophilic disease phase subgroup, D) vasculitic disease phase subgroup*

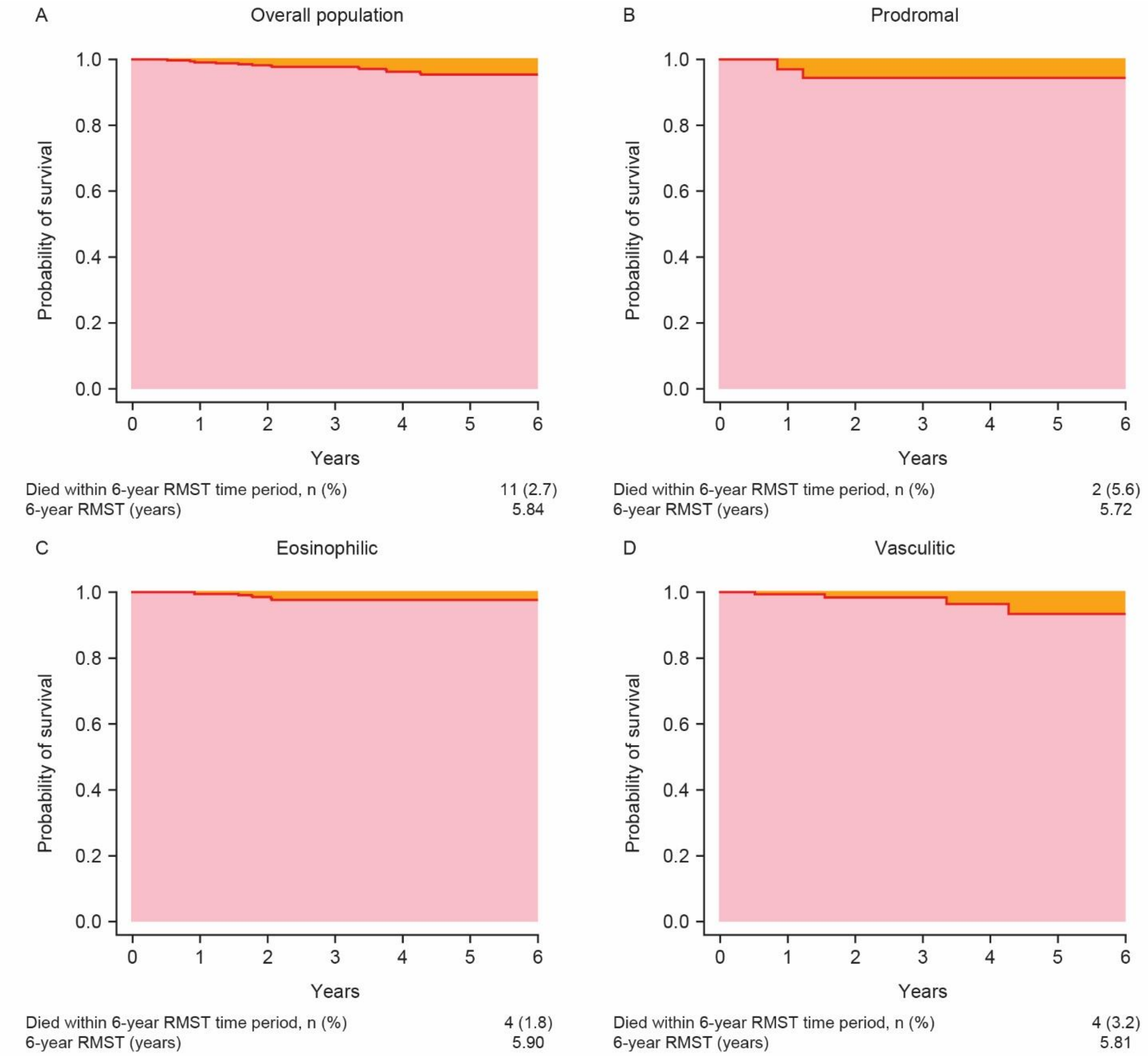

Overall survival was calculated from the date of EGPA diagnosis to death. Patients without record of death were censored at EOF or at 6 years after the diagnosis date, whichever came first. Patients with record of death were censored at 6 years after the diagnosis date if the duration between EGPA diagnosis and death was over 6 years. Because the incidence of death was observed to be <50%, the median survival time could not be estimated, and RMST was reported instead. RMST is a measure of the average EFS time and is estimated by the area under the KM curve during a specified period of time.

EFS, event-free survival; EGPA, eosinophilic granulomatosis with polyangiitis; EOF, end of follow-up; KM, Kaplan–Meier; RMST, restricted mean survival time.
